# Supplementary material for: Relationships between sensory processing patterns and metabolic risk factors among community dwelling people with metabolic syndrome: A cross-sectional and correlational research design
Source: PLoS One. 2024 Sep 6;19(9):e0308421. doi: 10.1371/journal.pone.0308421 (PMC11379226; doi:10.1371/journal.pone.0308421)
Supplement: S1 File — (PDF) [file pone.0308421.s001.pdf]

The Thai Sensory Patterns Assessment (TSPA)

The Thai Sensory Patterns Assessment is a self-report type of behavioral responses to 6 sensory stimuli: visual, auditory, smell and taste, vestibular and proprioceptive senses. In this assessment, the sensory processing patterns are divided into 2 types: the sensory preferences and the sensory arousals.

User Criteria.

1. General people over 15 years old.
2. Without cognitive impairment
3. Whoever able to read and write English.

Contents and Scoring

This assessment is a self-report of behavioral responses to sensory input in daily living and consists of 60 items, which are divided into two parts as follows:

1. The sensory preferences (Module I: 35 items) defined as the type of sensory stimulus that tends to make individuals feel affirmed and more comfortable, and is even pleasurable when receiving sensory input.
2. The sensory arousals (Module II: 25 items) defined as the levels of the nervous system's alertness to detect, register, and response to sensory input in daily life.

The user have to indicate how often they respond to the sensory event in everyday life by rating on a 5-Likert scale in each item (never, seldom, occasionally, frequently, and always). The assessment measured the frequency of response to sensory stimuli using the Likert scale from 1–5 and determined scoring for each item (1 = never, 2 = seldom, 3 = occasionally, 4 = frequently, 5 = always) in the module I: sensory preferences. While the module II: sensory arousals (1 = never, 2 = seldom, 3 = occasionally, 4 = frequently, 5 = always) in items with high arousal and (5 = never, 4 = seldom, 3 = occasionally, 2 = frequently, 1 = always) in items with low arousal.

The scores for each sensory modality were combined and reported separately as a percentage (below 25% = low, at 25-75% = moderate, and above 75% = high sensory preferences or levels of sensory arousals).

Psychometric properties

This assessment was develop in 2020 (Pomngen, Srikhamjak , & Putthinoi, 2020). The content validity was conducted by analyzing the index of item-objective congruence (IOC) from five experts. The construct validity was examined by confirmatory factor analysis and the internal consistency of their responses was assessed using coefficient-α among the total sample of 400 participants aged 15 years and over in Muang, Doi Saket and Mae On district, Chiang Mai province by multistage sampling, and 40 participants completed a retest after 1 week for test-retest reliability.

The result showed that the assessment consisted of 2 modules: module I: sensory preferences in 35 items and module II: sensory arousals in 25 items. Each module was divided into 6 categories according to the type of sensory modalities: visual, auditory, smell & taste, tactile, proprioceptive and vestibular senses. The content validity examinations the index of item-objective congruence (IOC) of the assessment module I and module II ranged from 0.60-1.00. The internal consistency reliability of assessment in the form of coefficient-α were 0.89 in module I and 0.62 in module II. The test-retest reliability with intraclass correlation coefficient method were 0.93 in module I and 0.77 in module II. The construct validity by confirmatory factor analysis was found that there were six factors in each module, and all of items had a high factor loading (more than a value of 0.4), including visual (factor loading of module I = 0.543-0.735, module II = 0.704-0.823), auditory (factor loading of module I = 0.422-0.807, module II = 0.484-0.770), smell & taste (factor loading of module I = 0.492-0.777, module II = 0.675-0.849), tactile (factor loading of module I = 0.627-0.754, module II = 0.510-0.760), proprioceptive (factor loading of module I = 0.519-0.815, module II = 0.723-0.745) and vestibular (factor loading of module I = 0.538-0.656, module II = 0.608-0.698) senses. In conclusion, the sensory patterns assessment had validity and reliability at an acceptable level.

Read and complete the following steps below.

1. Fill out personal information

Name: .... Sex: ❑ M ❑ F

Age: ....years Chronic disease: ....

Career: .... Date: ....

2. Taking the Self-Report Assessment

Read each question. Then select the answer by marking a cross (X) in the box corresponded to the frequency of the 6 sensory neurological response behaviors that are normal in daily life with the following criteria:

Never: never feel like or behave like this; the behavior shown only 5%

Seldom: rarely response to the stimuli; the behavior shown only 25%

Occasionally: feel like sometimes/this behavior happens at least once a week; the behavior shown 50%

Frequently: feel favorable almost always/this behavior happens at least once a day; the behavior shown 75%

Always: feel like it every time/this behavior happens at least several times a day.

Module I: Sensory Preferences

Sensory preferences defined as the type of sensory stimulus that registers most quickly, requires less energy when decoding sensory information and tends to make individuals feel affirmed and more comfortable, and is even pleasurable when receiving sensory input.

Read each item below. Mark a cross (X) in the box that reflects the frequency of your behavioral response in everyday life.

Visual sense

No.	Behavior	Never	Seldom	Occasionally	Frequently	Always
1.	Like it when people are around or like being in a crowded place	1	2	3	4	5
2.	Like to look at people or vehicles that pass by	1	2	3	4	5
3.	Like to dress or look at people dressed in bright colors	1	2	3	4	5
4.	Like sightseeing or like the places where have many colorful decorations	1	2	3	4	5
					Total scores	

Auditory sense

No.	Behavior	Never	Seldom	Occasionally	Frequently	Always
1.	Like listening to music	1	2	3	4	5
2.	Like listening to radios or televisions while working or doing activities, such as hobbies, leisure, activities, etc.	1	2	3	4	5
3.	Like listening to natural sounds	1	2	3	4	5
4.	Like to sing.	1	2	3	4	5
5.	Like to listen to melody.	1	2	3	4	5
					Total scores	

Smell and Taste senses						
No.	Behavior	Never	Seldom	Occasionally	Frequently	Always
1.	Like to use perfume or cologne	1	2	3	4	5
2.	Like to smell herbs, sniff drugs or snuff	1	2	3	4	5
3.	Like to smell food before eating	1	2	3	4	5
4.	Like eating sweets or smelling food	1	2	3	4	5
5.	Like to wear clothes or use a scented blanket	1	2	3	4	5
6.	Like to smell clothes, blankets, or other costumes before using them.	1	2	3	4	5
7.	Like to eat strong-flavored food.	1	2	3	4	5
8.	Like to taste exotic food or like finding delicious food	1	2	3	4	5
9.	Like to eat little things or snacks such as sweets, sweet drinks, soft drinks, tea, coffee, snacks, etc.	1	2	3	4	5
					Total scores	

Tactile sense						
No.	Behavior	Never	Seldom	Occasionally	Frequently	Always
1.	Like it when someone strokes the back, shoulders or head.	1	2	3	4	5
2.	Like sleeping in cuddling pillows, hugging dolls or blankets	1	2	3	4	5
3.	Like exposing to nature such as walking on bare feet or soaking in water when going to waterfalls, etc.	1	2	3	4	5
4.	Like a massage or a spa	1	2	3	4	5
					Total scores	

Vestibular sense						
No.	Behavior	Never	Seldom	Occasionally	Frequently	Always
1.	Enjoy acrobatic activities such as jumping from a height, trapeze, etc.	1	2	3	4	5
2.	Like climbing trees or going up to a high view point.	1	2	3	4	5
3.	Like to rock and move the head to the rhythm of the music	1	2	3	4	5
4.	Like sitting on rocking chairs, marry go rounds or cradle swings.	1	2	3	4	5
5.	Like to rock body while sitting unconsciously	1	2	3	4	5
6.	Like trapeze activities	1	2	3	4	5
7.	Prefer to do activities that involve turning the head and body, such as ballet, dance, etc.	1	2	3	4	5
8.	Like to ride on vehicles that have speed and change directions.	1	2	3	4	5
					Total scores	

Proprioceptive sense						
No.	Behavior	Never	Seldom	Occasionally	Frequently	Always
1.	Like to shake legs while sitting or knock on a table	1	2	3	4	5
2.	Like moving along with the rhythm of the music	1	2	3	4	5
3.	Prefer doing activities that involve pulling, pushing, banging, such as boxing, jumping rope, lifting weights, etc.	1	2	3	4	5
4.	Like to squeeze or crush objects with hands, such as kneading dough, kneading clay, etc.	1	2	3	4	5
5.	Like to rotate objects in hands such as pencils, pens, etc.	1	2	3	4	5
					Total scores	

Module II: Sensory arousals
**Sensory arousals** defined as the levels of the nervous system's alertness to detect, register, and response to sensory input in daily life.
Read each item below. Mark (X) in the box that reflects the frequency of your behavioral response in everyday life.

Visual sense						
No.	Behavior	Never	Seldom	Occasionally	Frequently	Always
1.	See fast changes in light in each day	1	2	3	4	5
2.	Immediately see when people approach	1	2	3	4	5
3.	Quickly see the change occurring to people around	1	2	3	4	5
					Total scores	

Auditory sense						
No.	Behavior	Never	Seldom	Occasionally	Frequently	Always
1.	Speak loudly.	5	4	3	2	1
2.	Have to let others call several times to realize	5	4	3	2	1
3.	Frequently and repeatedly ask while having conversations	5	4	3	2	1
4.	Cover ears with hands when listening	5	4	3	2	1
5.	Turn on the radio, television or communication device loudly	5	4	3	2	1
6.	Hard to tell or give a certain direction of the sound	5	4	3	2	1
					Total scores	

Smell and Taste senses						
No.	Behavior	Never	Seldom	Occasionally	Frequently	Always
1.	Can be able to eat food that has strong or pungent smell	5	4	3	2	1
2.	Can eat spicy food	5	4	3	2	1
3.	Can eat all kinds of food with no complaint	5	4	3	2	1
					Total scores	

Tactile sense						
No.	Behavior	Never	Seldom	Occasionally	Frequently	Always
1.	Easily feel hurt or irritated	1	2	3	4	5
2.	Feel sick or vomiting while brushing teeth	1	2	3	4	5
3.	Feel irritated with some fabrics or daily chemical products, such as dishwashing liquid, detergent, etc.	1	2	3	4	5
					Total scores	

Vestibular sense						
No.	Behavior	Never	Seldom	Occasionally	Frequently	Always
1.	Easily to have motion sickness or seasickness	1	2	3	4	5
2.	Feel dizzy easily when taking the elevator or escalator	1	2	3	4	5
3.	Feel dizzy when sitting on a crib or on a carousel	1	2	3	4	5
					Total scores	

Proprioceptive sense						
No.	Behavior	Never	Seldom	Occasionally	Frequently	Always
1.	Move awkwardly or clumsily	5	4	3	2	1
2.	Have inaccurate movement and cannot estimate moving distance precisely; always banging head when bending, falling down while stepping, etc.	5	4	3	2	1
3.	Often being complained about doing anything hard; playing hard, closing the door loudly	5	4	3	2	1
4.	Lose distance and cannot follow others when walking or running	5	4	3	2	1
5.	Often break or damage items because of excessive force while handling	5	4	3	2	1
6.	Put pressure until the paper is embossed or the pencil is broken while writing or painting	5	4	3	2	1
7.	Often being complained about walking with heavy bumps or a harsh sound	5	4	3	2	1
					Total scores	

Interpretation

Module I: Sensory Preferences		
Senses	$\frac{\textbf{Total raw score}}{\textbf{Maximum score}} \times 100$	Percentage (%)
Visual	$\frac{}{20} \times 100$	
Auditory	$\frac{}{25} \times 100$	
Smell-taste	$\frac{}{45} \times 100$	
Tactile	$\frac{}{20} \times 100$	
Vestibular	$\frac{}{40} \times 100$	
Proprioceptive	$\frac{}{25} \times 100$	
**Module II: Sensory arousals**		
Senses	$\frac{\textbf{Total raw score}}{\textbf{Maximum score}} \times 100$	Percentage (%)
Visual	$\frac{}{15} \times 100$	
Auditory	$\frac{}{30} \times 100$	
Smell-taste	$\frac{}{15} \times 100$	
Tactile	$\frac{}{15} \times 100$	
Vestibular	$\frac{}{15} \times 100$	
Proprioceptive	$\frac{}{35} \times 100$	

Result

Levels of sensory preferences: an increased percentage of sensory preferences in each sensory modality reflects what specific sensory stimuli individual prefer or tends to make individual feel affirmed and more comfortable, while a decreased percentage of sensory preferences means that individual tend to not prefer or avoid the sensory input. In this assessment, the levels of sensory preferences are divided into 3 levels as below:

- Low levels of sensory preference = less than 25%
- Moderate levels of sensory preference = 25% - 75%
- High sensory preference = more than 75%

Levels of sensory arousals: the percentage of sensory arousals reflects how individual's nervous system notice sensory stimuli in daily living. An increased percentage of sensory arousals in each sensory modality reflects what specific sensory stimuli individual tends to notice, detect, register, and response quickly, and people with higher levels of sensory arousals trend to sensitive to or being overwhelmed by sensory input in daily living, while a decreased percentage of sensory arousals means that individual tend to miss things that others notice or need more sensory input to notice what is going on. In this assessment, the levels of sensory arousals are divided into 3 levels as below:

- Low levels of sensory arousal = less than 25%
- Moderate levels of sensory arousal = 25% - 75%
- High levels of sensory arousal = more than 75%

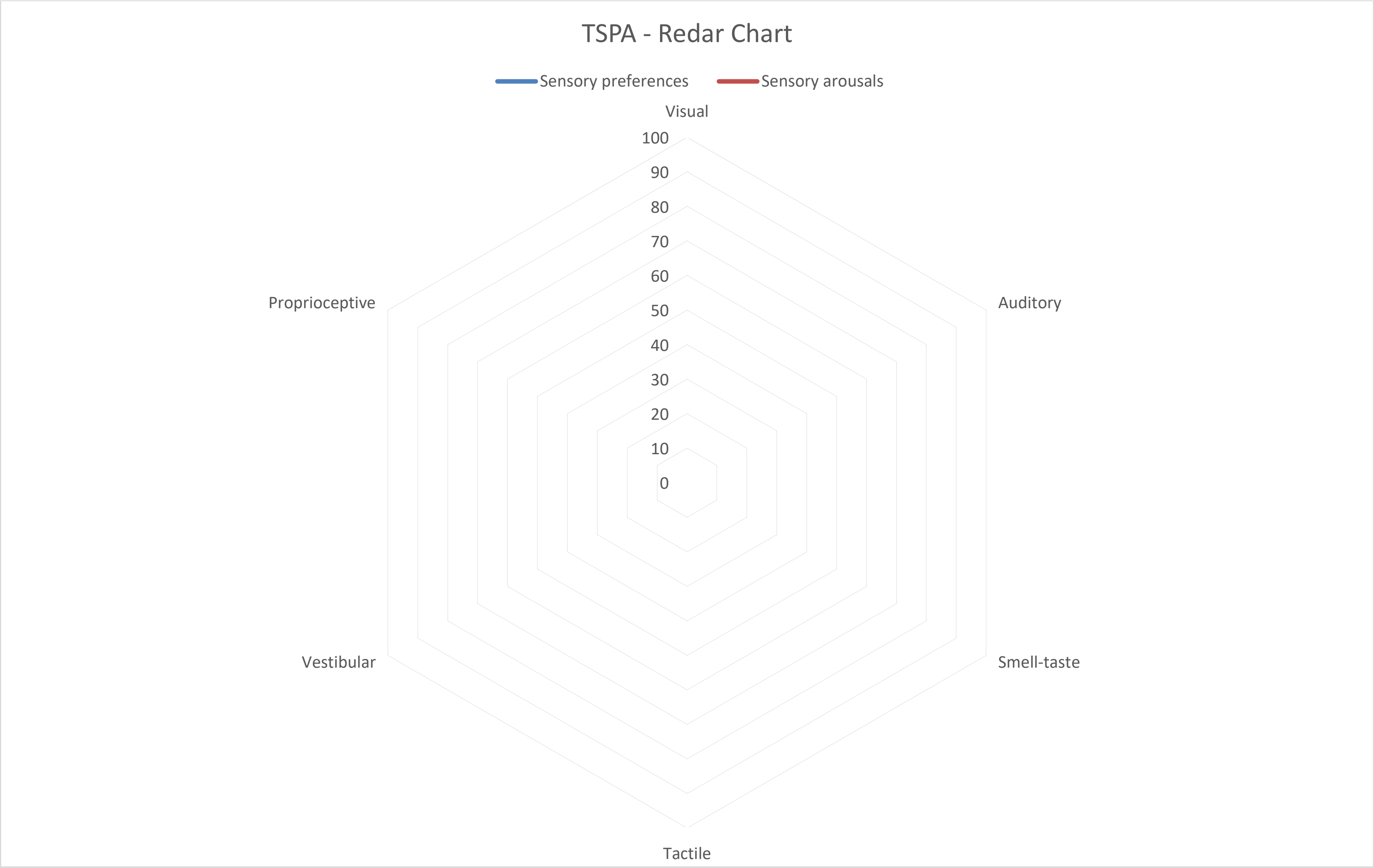
TSPA - Redar Chart
Sensory preferences
Sensory arousals
Visual
Auditory
Smell-taste
Tactile
Vestibular
Proprioceptive
100
90
80
70
60
50
40
30
20
10
0

Reference:

Pomngen I, Srikhamjak T, Putthinoi S. Development of the Sensory Patterns Assessment. M. Sc. Thesis, Chiang Mai University. 2020.
