# Supplementary material for: Relationships between sensory processing patterns and metabolic risk factors among community dwelling people with metabolic syndrome: A cross-sectional and correlational research design
Source: PLoS One. 2024 Sep 6;19(9):e0308421. doi: 10.1371/journal.pone.0308421 (PMC11379226; doi:10.1371/journal.pone.0308421)
Supplement: S2 File — (PDF) [file pone.0308421.s002.pdf]

STROBE Statement—Checklist of items that should be included in reports of ***cross-sectional studies***

	Item No	Recommendation
**Title and abstract**	1	(*a*) Indicate the study's design with a commonly used term in the title or the abstract<br>✓ in Line 2-3
		(*b*) Provide in the abstract an informative and balanced summary of what was done and what was found<br>✓ in Line 25-54
**Introduction**		
Background/rationale	2	Explain the scientific background and rationale for the investigation being reported<br>✓ in Line 60-183
Objectives	3	State specific objectives, including any prespecified hypotheses<br>✓ in Line 177-183
**Methods**		
Study design	4	Present key elements of study design early in the paper<br>✓ in Line 185-191
Setting	5	Describe the setting, locations, and relevant dates, including periods of recruitment, exposure, follow-up, and data collection<br>✓ in Line 201-203
Participants	6	(*a*) Give the eligibility criteria, and the sources and methods of selection of participants<br>✓ in Line 208-224, 225-252
Variables	7	Clearly define all outcomes, exposures, predictors, potential confounders, and effect modifiers. Give diagnostic criteria, if applicable<br>✓ in Line 323-326
Data sources/ measurement	8*	For each variable of interest, give sources of data and details of methods of assessment (measurement). Describe comparability of assessment methods if there is more than one group<br>✓ in Line 253-309
Bias	9	Describe any efforts to address potential sources of bias<br>✓ in Line 258-259 and Line 305-309
Study size	10	Explain how the study size was arrived at<br>✓ in Line 199-200
Quantitative variables	11	Explain how quantitative variables were handled in the analyses. If applicable, describe which groupings were chosen and why<br>✓ in Line 312-327
Statistical methods	12	(*a*) Describe all statistical methods, including those used to control for confounding<br>✓ in Line 310-327
		(*b*) Describe any methods used to examine subgroups and interactions<br>✓ in Line 317-318
		(*c*) Explain how missing data were addressed<br>There was no missing data
		(*d*) If applicable, describe analytical methods taking account of sampling strategy<br>✓ in Line 229-237
		(*e*) Describe any sensitivity analyses<br>No sensitivity analyses

**Results**		
Participants	13*	(a) Report numbers of individuals at each stage of study—eg numbers potentially eligible, examined for eligibility, confirmed eligible, included in the study, completing follow-up, and analysed<br>✓ in Line 329-334
		(b) Give reasons for non-participation at each stage<br>✓ in Line 332-333
		(c) Consider use of a flow diagram
Descriptive data	14*	(a) Give characteristics of study participants (eg demographic, clinical, social) and information on exposures and potential confounders<br>✓ in Line 335-349 and in Table 1-2
		(b) Indicate number of participants with missing data for each variable of interest<br>✓ in Line 333-334
Outcome data	15*	Report numbers of outcome events or summary measures<br>✓ in Line 343-349, 355-362, 367-375, 381-388
Main results	16	(*a*) Give unadjusted estimates and, if applicable, confounder-adjusted estimates and their precision (eg, 95% confidence interval). Make clear which confounders were adjusted for and why they were included<br>✓ Table 5 in line 389 (95% confidence interval)
		(*b*) Report category boundaries when continuous variables were categorized<br>✓ sensory patterns in line 343-349 (metabolic risk variables), 355-362 (sensory processing patterns), 367-375 and 381-389 (correlation between sensory patterns and metabolic risk factors)
		(*c*) If relevant, consider translating estimates of relative risk into absolute risk for a meaningful time period<br>✓ in line 203 during January 1st, 2023 to April 30th, 2023, there was no risk for a meaningful time period
Other analyses	17	Report other analyses done—eg analyses of subgroups and interactions, and sensitivity analyses<br>✓ in Line 343-349
**Discussion**		
Key results	18	Summarise key results with reference to study objectives<br>✓ in Line 394-400
Limitations	19	Discuss limitations of the study, taking into account sources of potential bias or imprecision. Discuss both direction and magnitude of any potential bias<br>✓ in Line 532-536
Interpretation	20	Give a cautious overall interpretation of results considering objectives, limitations, multiplicity of analyses, results from similar studies, and other relevant evidence<br>✓ in Line 525-532
Generalisability	21	Discuss the generalisability (external validity) of the study results<br>✓ in Line 532-539
**Other information**		
Funding	22	Give the source of funding and the role of the funders for the present study and, if applicable, for the original study on which the present article is based<br>This research was supported by the Department of Occupational Therapy, Faculty of Associated Medical Sciences, Chiang Mai University under Grant (AMS-2023).

*Give information separately for exposed and unexposed groups.

Note: An Explanation and Elaboration article discusses each checklist item and gives methodological background and published examples of transparent reporting. The STROBE checklist is best used in conjunction with this article (freely available on the Web sites of PLoS Medicine at http://www.plosmedicine.org/, Annals of Internal Medicine at http://www.annals.org/, and Epidemiology at http://www.epidem.com/). Information on the STROBE Initiative is available at www.strobe-statement.org.
