# Supplementary material for: Relationships between sensory processing patterns and metabolic risk factors among community dwelling people with metabolic syndrome: A cross-sectional and correlational research design
Source: PLoS One. 2024 Sep 6;19(9):e0308421. doi: 10.1371/journal.pone.0308421 (PMC11379226; doi:10.1371/journal.pone.0308421)
Supplement: S3 File — (PDF) [file pone.0308421.s003.pdf]

FBG	SBP	DBP	WC	P-visual	P-auditory	P-smell-taste	P-tactile	P-vestibular
100	138	85	90	20	40	39.96	30	20
147	127	97	82	20	20	44.4	35	42.5
116	149	97	103	30	32	62.16	40	20
110	140	85	90	30	56	35.52	20	20
124	135	85	88	30	76	68.82	45	22.5
100	130	85	87	30	40	46.62	20	20
142	130	90	80	30	92	48.84	20	30
100	132	85	87	30	44	59.94	30	20
116	135	85	98	30	88	57.72	60	42.5
100	133	85	80	30	48	48.84	30	20
125	130	87	89	30	48	48.84	30	20
114	141	89	90	35	48	59.94	40	22.5
105	130	85	90	40	60	51.06	40	30
110	131	85	98	40	48	53.28	35	32.5
172	153	89	80	40	64	53.28	40	25
100	134	89	80	40	60	51.06	55	40
100	137	85	87	40	48	48.84	55	32.5
132	150	85	90	40	68	28.86	35	35
100	140	92	91	40	48	53.28	30	22.5
101	138	86	94	40	80	73.26	45	30
105	150	89	89	40	56	51.06	35	25
128	132	91	86	45	72	39.96	20	42.5
110	132	85	80	45	60	48.84	45	45
120	137	85	114	45	52	46.67	30	22.5
103	132	85	80	45	44	39.96	25	20
100	130	90	90	45	72	53.28	40	25
120	130	90	80	45	52	48.84	30	20
128	130	85	80	45	44	44.4	20	27
106	147	87	87	45	68	62.16	20	27.5
104	143	90	89	45	64	51.11	30	32.5
125	135	95	101	45	64	39.96	65	60
109	135	85	112	45	40	77.7	50	20
137	130	90	89	50	60	73.26	40	40
157	135	95	110	50	60	57.72	55	40
100	135	85	82	50	60	57.72	45	40
135	130	90	98	50	72	66.66	60	35
106	150	90	101	50	56	59.94	30	45
100	140	85	100	50	64	60	50	37
120	130	90	84	50	60	57.72	30	27.5
105	130	90	102	50	84	62.16	45	50
100	130	85	84	50	60	64.38	70	40
100	130	85	84	50	64	42.18	50	37.5
100	130	85	95	50	44	60	30	20
99	134	85	90	50	92	71.04	60	35
100	130	85	106	55	100	46.67	70	20
125	135	85	87	55	32	64.38	45	25
138	130	85	98	55	56	59.94	25	20
100	126	90	80	55	72	73.26	80	60
100	130	85	90	55	72	44.4	20	25
114	135	90	94	55	64	62.16	30	40

100	137	85	87	55	56	37.74	30	30
139	130	90	92	55	64	46.62	40	47.5
172	130	90	95	55	72	42.18	35	42.5
101	149	85	80	55	48	44.4	45	20
104	138	85	100	55	72	62.16	50	37.5
125	130	90	102	55	68	53.28	25	25
125	135	86	87	60	48	59.94	30	32.5
100	143	95	88	60	60	42.18	35	22.5
151	130	90	100	60	60	57.72	60	57.5
110	141	85	108	60	72	48.84	25	22.5
100	130	90	84	60	48	66.6	60	32.5
100	130	85	92	60	52	37.74	40	25
123	136	87	90	60	84	62.16	35	37.5
109	130	90	95	60	80	59.94	40	45
126	135	90	90	60	100	48.84	60	60
100	128	79	93	60	60	60	60	20
100	140	80	92	60	60	60	60	20
100	130	90	90	60	80	60	30	25
102	136	90	96	60	72	75.48	30	30
101	130	80	81	60	92	73.26	55	40
135	135	87	82	60	68	57.72	25	30
138	137	85	91	60	76	62.16	40	27.5
169	135	90	80	60	60	60	60	60
100	130	80	89	60	72	66.6	65	50
102	135	86	101	60	72	66.6	65	50
140	130	85	92	60	68	53.28	25	30
100	130	90	84	60	100	86.58	100	60
100	135	85	107	64	51.06	35	30	40
139	134	85	98	65	80	55.5	25	35
129	135	90	87	65	56	53.28	55	20
100	130	85	90	65	64	59.94	50	37.5
124	130	85	132	65	92	68.82	45	42.5
100	130	90	90	65	80	68.82	70	35
100	140	88	84	65	64	42.18	25	27.5
105	132	86	90	65	64	42.18	25	27.5
140	130	85	81	65	64	42.18	25	27.5
116	130	85	90	65	64	42.18	25	27.5
116	145	85	94	65	88	64.38	60	37.5
100	130	85	81	65	72	66.6	55	27.5
100	140	85	94	65	56	42.18	20	20
120	135	85	90	70	100	59.94	40	32.5
103	135	85	90	70	68	70	60	60
132	140	85	92	70	76	62.16	50	37.5
112	135	90	84	70	76	62.16	45	35
119	135	90	94	70	60	68.82	50	27.5
102	130	80	102	70	60	66.6	55	45
119	155	85	81	70	76	57.72	30	30
161	135	90	112	70	100	84.36	35	32.5
100	130	85	90	75	84	62.16	60	47.5
104	130	85	88	75	88	73.26	50	30
101	150	90	95	75	64	59.94	60	32.5

148	135	85	91	75	68	71.04	45	40
108	138	85	94	75	64	64.38	25	49.5
101	135	86	93	75	100	64.38	35	30
127	138	85	99	75	100	57.72	40	30
100	130	85	90	80	68	75.48	50	42.5
100	145	85	87	80	32	53.28	55	25
100	135	80	80	85	88	88.8	70	50
110	130	85	84	85	80	66.6	50	32.5
147	133	89	80	90	92	53.28	20	20
122	164	87	84	90	84	59.94	50	22.5
125	132	86	82	90	76	66.6	60	47.5
115	130	85	98	90	48	68.82	40	20
103	135	85	80	90	84	66.6	50	20
145	130	85	100	95	92	86.58	45	27.5
114	135	90	126	95	84	62.16	60	40
140	130	85	88	95	100	79.92	65	32.5

P-proprioceptive	A-visaul	A-auditory	A-smell-taste	A-tactile	A-vestibular	A-proprioceptive
32	20	96.66	100	26.67	20	91.42
28	53.33	86.67	66.67	20	20	100
20	20	93.33	53.33	40	46.67	100
20	33.33	63.33	86.67	46.66	40	42.85
24	46.67	53.33	33.33	53.33	60	85.71
20	60	86.67	80	20	20	97.14
28	73.33	60	80	26.67	20	74.28
20	80	96.67	73.33	26.67	60	94.28
40	100	83	20	33.33	33.33	85.71
28	100	96.67	73.33	33.33	20	88.57
28	100	96.67	73.33	33.33	20	88.57
36	40	73.33	80	53	20	82.85
36	46.67	93.33	73.33	20	20	94.28
36	46.67	76.67	60	20	46.67	85.71
20	60	73.33	80	33.33	20	74.28
60	66.67	60	60	60	60	74.28
40	66.67	76.66	73.33	40	60	74.28
40	66.7	89.91	80.04	60.03	20	88.35
32	73.33	60	60	20	20	68.57
36	86.67	76.67	66.67	33.33	33.33	85.71
44	93.33	80	53.33	33.33	20	82.85
28	20	73.33	80	33.33	40	74.28
24	33.33	83.33	93.33	33.33	20	80
36	40	80	73.33	33.33	40	88.57
24	40	76.66	86.67	60	46.67	71.42
36	40	83.33	66.67	33.33	40	71.14
28	60	80	73.33	46.67	40	85.71
40	60	70	86.67	46.67	46.67	71.42
36	66.67	96.67	40	26.67	73.33	88.57
28	86.67	73.33	60	33.33	33.33	40
44	93.33	43.33	93.33	60	20	71.42
20	100	70	80	100	33.33	88.57
48	40	80	80	60	60	77.14
44	40	63.33	80	33.33	33.33	65.71
44	53.33	80	73.33	40	40	77.14
36	60	80	26.67	66.67	46.67	82.85
52	60	83.33	53.33	26.67	26.67	80
48	60	80	33.33	53.33	40	77.14
32	80	83.33	60	40	40	71.42
56	80	83.33	60	40	46.67	80
48	80	83.33	60	46.67	26.67	94.28
48	80	80	33.33	53.33	40	77.14
20	80	100	73.33	33.33	60	91.42
36	100	83.33	20	100	33.33	100
20	20	100	100	20	26.67	100
20	26.67	76.67	66.67	46.67	80	77.14
28	53.33	76.66	66.67	46.67	40	77.14
56	53.33	66.67	46.67	40	40	65.71
40	60	96.67	66.67	20	20	94.28
44	73.33	83.33	40	53.33	40	88.57

44	80	83.33	66.67	33.33	40	74.28
48	80	83.33	66.67	26.67	40	82.85
56	80	80	73.33	40	40	82.85
20	80	86.67	73.33	46.67	20	62.85
52	100	96.67	86.67	26.67	26.67	97.14
28	100	90	33.33	60	73.33	82.85
28	33.33	77.14	93.33	20	33.33	97.14
24	40	83.33	73.33	53.33	53.33	91.42
60	40	80	60	40	40	74.28
40	53.33	73.33	66.67	33.33	20	68.57
36	53.33	70	73.33	46.67	40	74.28
28	53.33	73.33	73.33	40	33.33	60
36	60	83.33	40	40	33.33	100
40	60	83.33	60	20	20	100
60	60	60	60	60	60	48.57
20	60	90	60	20	20	82.85
20	60	90	60	20	20	82.85
60	60	60	60	60	40	60
24	66.67	100	46.67	40	40	94.28
44	73.33	70	60	66.67	40	74.28
48	80	76.67	66.67	46.67	20	97.14
32	80	96.67	93.33	26.67	33.33	97.14
40	80	60	60	60	60	60
64	80	70	60	33.33	20	74.28
64	80	70	60	33.33	20	74.28
36	80.04	79.92	66.7	33.35	20	100
60	100	20	20	20	20	100
80	90	46.67	33.33	33.33	77.14	64
44	73.33	83.33	60	53.33	33.33	80
24	80	80	80	32	40	85.71
48	80	80	26.67	53.33	40	94.28
36	86.67	90	60	20	26.67	94.28
40	86.67	93.33	73.33	33.33	33.33	88.57
24	93.33	73.33	53.33	46.67	60	40
24	93.33	73.33	53.33	46.67	60	40
24	93.33	73.33	53.33	46.67	60	40
24	93.33	73.33	53.33	46.67	60	40
32	93.33	80	46.67	33.33	33.33	94.28
36	93.33	100	86.67	46.67	26.67	94.28
20	100	86.67	53.33	26.67	46.67	94.28
64	60	80	46.6	33.33	20	74.28
60	60	76.67	40	33.33	20	91.42
44	73.33	73.33	53.33	73.33	46.67	85.71
44	73.33	76.67	53.33	73.33	46.67	88.57
28	80	83.33	53.33	46.67	53.33	82.85
52	80	80	20	66.67	26.67	85.71
32	93.33	86.67	66	46.67	46.67	85.71
60	93.33	60	33.33	73.33	60	77.14
52	73.33	66.67	53.33	53.33	100	88.57
48	73.33	66.67	40	60	60	91.42
48	80	83.33	73.33	40	26.67	97.14

32	86.67	83.33	40	33.33	33.33	94.28
48	93.33	86.67	46.67	26.67	40	80
32	100	93.33	26.67	33.33	20	97.14
36	100	86.67	60	20	40	80
56	73.33	66.67	60	66.67	33.33	82.85
32	93.33	76.67	66.67	53.33	100	65.71
56	73.33	63.33	60	73.33	73.33	77.14
32	80	76.67	46.67	20	20	97.14
32	46.67	50	73.33	33.33	66.67	88.57
24	80	86.67	73.33	46.67	26.67	94.28
52	80	63.27	60.03	46.67	20	82.85
28	80	60	80	40	33.33	74.28
32	86.67	66.67	93.33	53.33	40	85.71
32	80.04	73.33	46.67	40	80	77.14
48	93.33	96.67	60	26.67	26.67	91.43
56	100	76.67	26.67	73.33	60	85.71
