# Supplementary material for: Relationships between sensory processing patterns and metabolic risk factors among community dwelling people with metabolic syndrome: A cross-sectional and correlational research design
Source: PLoS One. 2024 Sep 6;19(9):e0308421. doi: 10.1371/journal.pone.0308421 (PMC11379226; doi:10.1371/journal.pone.0308421)
Supplement: Attachment — Submitted filename: Response to Editor.docx [file pone.0308421.s004.docx]

Response to combined reviewers and editor

We greatly appreciate your thoughtful comments concerning our manuscript entitled "Sensory processing patterns as internal factors determining metabolic risk factors among community dwelling people with Metabolic Syndrome" (Manuscript ID: PONE-D-24-06360). Those comments are all valuable and very helpful for revising and improving our paper. We have studied comments carefully and have made correction which we hope meet with approval. Revised portion are marked in yellow highlighting in the paper and indicated by blue text.

The major points in the paper and the responds to the reviewers and editor comments are as flowing:

**Point 1:** The non-compliance with STROBE guidelines for cross-sectional studies. Note that this is a requirement for publication in Plos ONE (https://journals.plos.org/plosone/s/submission-guidelines#loc-guidelines-for-specific-study-types):

Response 1: We are thankful with your comments and suggestion regarding the non-compliance with STROBE guidelines for cross-sectional studies. We have carefully reviewed our manuscript to ensure it meets the requirements for publication by utilizing the STROBE guidelines for cross-sectional studies. Additionally, we have included a checklist indicating the lines where the corresponding items are clearly addressed and have uploaded this checklist as supplemental material.

**Point 2:** Both reviewers and I have concerns regarding the TSPA, which we cannot access due to language barriers (it is apparently written in Thai language) and the requirement of institutional access to see the relevant documentation. I am requesting that you include the English translation of the TSPA as supplemental material, and clarify on its content, reliability and the rationale behind its use.

**Response 2:** We truly appreciated your comments and suggestion about the Thai Sensory Patterns Assessment-adult version (TSPA). We included the English translation for the Thai Sensory Patterns Assessment-adult version (TSPA), thesis abstract - English version, and related information that help to clarify on its content, constructs, validity, reliability, administration and scoring procedures, results from factor analysis for the assessment as supplemental material.

**Point 3:** One reviewer requires more appropriate data analysis and reevaluating the Discussion based on this, and I wish to highlight this requirement as well, together with the recommendation made by the other reviewer, who is an expert in medical sciences, to perform a descriptive analysis grouping variables according to categories such as prediabetes/diabetes, hypertension stage 1/stage 2, etc.

**Response 3:** We truly appreciate your valuable comments regarding the need for more appropriate data analysis and performing a descriptive analysis by grouping variables according to categories such as prediabetes/diabetes, hypertension stage 1/stage 2, etc. In response to the reviewers' suggestions, we have changed the statistical analysis method from Spearman correlation to Kendall’s tau and from Kernel regression to General Linear Mixed Model, both of which are more suitable for non-parametric or non-normally distributed data. Furthermore, we have revised and updated the discussion section accordingly, now located on pages 19-24, lines 394-524. Additionally, we have addressed the information regarding metabolic variables by grouping variables into categories such as prediabetes/diabetes, hypertension stage 1/stage 2, etc., as suggested, on pages 15-16, lines 344-355.

**Point 4:** I ask you to do a careful revision of the manuscript in terms of grammar and language. For example, in lines 231-233 you say that "there were high arousal levels...except for propioceptive and auditory senses", while Table 2 shows the opposite**.**

**Response 4:** We truly appreciate and agree with your comments regarding the manuscript’s grammar and language. After carefully revising the manuscript based on your feedback and the suggestions of the two reviewers, we have sent it to an English specialist who is a native English speaker for further revision. Additionally, we have a copy of the manuscript showing track changes by a native English as supporting information.

Special thanks to you for your good comments and suggestion. We tried our best to improve the manuscript and made some changes in the manuscript.

We appreciate for editor and reviewers’ warm work earnestly and hope that the correction will meet with approval. Once again, thank you very much indeed for your comments and suggestions.

Best regards,

Ilada Pomngen and research team
