# Supplementary material for: Relationships between sensory processing patterns and metabolic risk factors among community dwelling people with metabolic syndrome: A cross-sectional and correlational research design
Source: PLoS One. 2024 Sep 6;19(9):e0308421. doi: 10.1371/journal.pone.0308421 (PMC11379226; doi:10.1371/journal.pone.0308421)
Supplement: Attachment — Submitted filename: Response to Editor.docx [file pone.0308421.s005.docx]

Response (round-2) to editor

We greatly appreciate your thoughtful comments concerning our manuscript entitled "Sensory processing patterns as internal factors determining metabolic risk factors among community dwelling people with Metabolic Syndrome" (Manuscript ID: PONE-D-24-06360R1). Those comments are all valuable and very helpful for revising and improving our paper. We have studied comments carefully and have made correction which we hope meet with approval. Revised portion are marked in yellow highlighting in the paper and indicated by blue text.

The major points in the paper and the responds to the reviewers and editor comments are as flowing:

**Point 1:** The GLMM needs work. Please report the family and link function used in the model and why. Report the model fit statistics, including the null models. Please report the software used to conduct the analyses.

Response 1: We truly appreciate your comments regarding the GLMM. Following your suggestion, we have now added the information regarding the GLMM analysis, including the family, link function, model fit statistics and software used to conduct the analyses. Now, the revision is on page 14, lines 324-331.

**Point 2:** Report the specific test used for the power calculation in G*Power

**Response 2:** We appreciate on your comments and suggestions. We have added the specific test for the power calculation in G*Power on page 8, lines 194-195.

**Point 3:** Be careful not to overstretch the results, given that the correlations you obtained are weak, for example when you speak of “unexpected results” on relationship between WC and auditory arousal.

**Response 3:** We would like to thank you for your comments and suggestion. We have carefully revised the manuscript and rephased this part, ensuring not to overstretch the interpretation of the results. Now, it is on the page 22, line 473-478.

**Point 4:** Please revise the “other comments” at the end of review 1 and the minor points made by reviewer 2.

**Response 4:** We truly appreciate and agree with your and reviewers’ comments. We have carefully revised all of the feedback and suggestions from both reviewers.

**Point 5:** Please do a new English check. I have seen the language check document you attached, but new mistakes appeared, such as “The example of items…” (line 289), “For psychometric properties. The…” (line 297), line 304 should be 0.849, lines 408 to 411, and several others. Avoid anthropomorphizing, as reviewer 1 says, for example “Kendall’s Tau found” in the abstract.

**Response 5:** We truly appreciate and agree with your comments regarding the manuscript’s grammar and language. After carefully revising the manuscript based on your feedback and the suggestions of the two reviewers, we have sent it again to an English specialist who is a native English speaker for further revision. Additionally, we have a copy of the manuscript showing track changes by a native English as supporting information.

- “The examples of items…” (line 284)
- “For psychometric properties. The…” (line 292)
- “0.849” (line 299)
- line 412-414
- the abstract (line 38 and 42)

**Point 6:** Line 440: should it be tactile sense?

**Response 6:** Thank you for your comment. We have reviewed the manuscript and confirmed that the use of the term "tactile sense" in line 440 is correct, and this finding showed in Table 5, which analyzed by the GLMM. However, to avoid any confusion, we have added an explanation in the manuscript referencing the specific statistics that support the significance to provide clearer understanding for the readers. Now, the revision is on page 21, line 445.

Special thanks to you for your good comments and suggestion. We tried our best to improve the manuscript and made some changes in the manuscript.

We appreciate for editor and reviewers’ warm work earnestly and hope that the correction will meet with approval. Once again, thank you very much indeed for your comments and suggestions.

Best regards,

Ilada Pomngen and research team
